# Kinin B1 receptor: a potential therapeutic target in sepsis-induced vascular hyperpermeability

**DOI:** 10.1186/s12967-020-02342-8

**Published:** 2020-04-19

**Authors:** Stéphanie Ruiz, Fanny Vardon-Bounes, Marie Buléon, Céline Guilbeau-Frugier, Marie-Hélène Séguelas, Jean-Marie Conil, Jean-Pierre Girolami, Ivan Tack, Vincent Minville

**Affiliations:** 1 Department of Anesthesiology and Intensive Care, Rangueil Hospital-University Hospital of Toulouse, 1 Avenue du Professeur Jean Poulhès TSA 50032, 31059 Toulouse Cedex 9, France; 2 Institute of Metabolic and Cardiovascular Diseases, INSERM/UPS UMR, 1048-I2MC, Equipe 3, Paul Sabatier University, Toulouse, France; 3 Department of Forensic Medicine, Rangueil Hospital-University Hospital of Toulouse, 1 Avenue du Professeur Jean Poulhès TSA 50032, 31059 Toulouse Cedex 9, France; 4 Biological Electron Microscopy Center, Rangueil Faculty of Medicine, Toulouse University, Toulouse, France; 5 Department of Physiology, Rangueil Hospital–University Hospital of Toulouse, 1 Avenue du Professeur Jean Poulhès TSA 50032, 31059 Toulouse Cedex 9, France

**Keywords:** Septic shock, Endothelial permeability, VE-cadherin, Kinin B1 receptor, B1R antagonist R-954

## Abstract

**Background:**

In sepsis, the endothelial barrier becomes incompetent, with the leaking of plasma into interstitial tissues. VE-cadherin, an adherens junction protein, is the gatekeeper of endothelial cohesion. Kinins, released during sepsis, induce vascular leakage and vasodilation. They act via two G-protein coupled receptors: B1 (B1R) and B2 (B2R). B1R is inducible in the presence of pro-inflammatory cytokines, endotoxins or after tissue injury. It acts at a later stage of sepsis and elicits a sustained inflammatory response. The aim of our study was to investigate the relationships between B1R and VE-cadherin destabilization in vivo in a later phase of sepsis.

**Methods:**

Experimental, prospective study in a university research laboratory. We used a polymicrobial model of septic shock by cecal ligation and puncture in C57BL6 male mice or C57BL6 male mice that received a specific B1R antagonist (R-954). We studied the influence of B1R on sepsis-induced vascular permeability 30 h after surgery for several organs, and VE-cadherin expression in the lung and kidneys by injecting R-954 just before surgery. The 96-h survival was determined in mice without treatment or in animals receiving R-954 as a “prophylactic” regimen (a subcutaneous injection of 200 µg/kg, prior to CLP and 24 h after CLP), or as a “curative” regimen (injection of 100 µg/kg at H6, H24 and H48 post-surgery).

**Results:**

B1R inactivation helps to maintain MAP above 65 mmHg but induces different permeability profiles depending on whether or not organ perfusion is autoregulated. In our model, VE-cadherin was destabilized in vivo during septic shock. At a late stage of sepsis, the B1R blockade reduced the VE-cadherin disruption by limiting eNOS activation. The survival rate for mice that received R-954 after sepsis induction was higher than in animals that received an antagonist as a prophylactic treatment.

**Conclusions:**

B1R antagonizing reduced mortality in our model of murine septic shock by limiting the vascular permeability induced by VE-cadherin destabilization through maintenance of the macrohemodynamics, consequently limiting organ dysfunctions.

## Background

Sepsis and septic shock remain leading causes of high morbimortality related to organ dysfunctions [1, 2]. Epithelial and endothelial barriers support homeostasis and organ function by establishing gradients between each compartment [3]. In sepsis, the endothelial barrier becomes incompetent, with plasma leakage into interstitial tissues resulting in edema which contributes to hypovolemia and poor tissue perfusion.

The endothelium drives the host–pathogen responses by promoting inflammation, coagulation and vascular permeability, through activation of the contact-phase system and the kinin-kallikrein system. When this response is uncontrolled, organ failures occur. Kinins, released at the early stage of sepsis, induce vascular leakage and vasodilation. They act via two G-protein coupled receptors: B1 (B1R) and B2 (B2R). The constitutive B2R mediates the action of kinins immediately after a harmful stimulus during the acute phase of inflammation. B1R is inducible in the presence of pro-inflammatory cytokines, endotoxins or after tissue injury. It acts at a later stage and elicits a sustained inflammatory response [4–6].

Endothelial cohesion is ensured by an adherens junction protein, Vascular Endothelial cadherin (VE-cadherin). When VE-cadherin is destabilized, the endothelium loses its barrier function. The role of this cadherin in sepsis-induced hyperpermeability has been poorly investigated in vivo in a polymicrobial sepsis model. However, studies on cell cultures or after lipopolysaccharide (LPS) challenge tend to confirm its pivotal action in the pathophysiology of endothelial dysfunction [7, 8]. Previous studies show how difficult it is to control the regulation of the sepsis-induced inflammatory response during the initial phase. However, it would be interesting to limit the chronicization of endothelial dysfunction in order to optimize septic shock management.

With this in mind, this study investigates the effects in vivo of a B1R antagonist, the R-954, on septic hyperpermeability, VE-cadherin destabilization and survival in a later phase of polymicrobial sepsis in mice induced by cecal ligation and puncture (CLP).

## Methods

### Animals

The animals used in this study were 15 to 25-week old male mice, wild-type strain C57BL/6J (WT). Animal experimentation was conducted according to national and institutional animal care and ethical guidelines and was approved by the local board. Sepsis was induced by cecal ligation and puncture (CLP) as previously described, with a cecal ligation height of 20% and a 20G needle [9]. Animals were anesthetized with an intraperitoneal injection of ketamine and xylazine (250 mg/kg and 10 mg/kg, respectively). Immediately post-surgery, 1 ml of saline was administered subcutaneously for fluid resuscitation. Pain control for CLP and sham mice was achieved with 0.05 mg/kg buprenorphine every 12 h. Mice were housed in a temperature-controlled room on a 12-h light–dark cycle and had access to water and food ad libitum.

### Experimental protocol

The flow charts of the three experimental series are provided in the Additional file 1.

#### First experimental series: role of B1R in sepsis-induced late vascular permeability

We first examined the influence of B1R on sepsis-induced vascular permeability of the heart, lungs, kidneys, liver and small intestine, 30 h after surgery, using the Evans blue dye (EBD) extravasation method (0.5 g/100 ml, Assistance Publique-Hôpitaux de Paris pharmacy), as previously described [10]. This timing corresponds to a “later” phase of sepsis and refers to the period following the early hyperinflammatory phase. It corresponds to a late innate immune dysfunction. In the cecal ligation and puncture model, this phase occurs at least 20 h after surgery in mice [9, 11].

The animals were divided into 3 groups: sham-operated C57BL/6J controls (WT sham; n = 8), CLP-induced septic C57BL/6J mice (WT CLP; n = 6) and CLP-induced septic C57BL/6J mice that received prophylactic B1R antagonist R-954 (WT CLP R-954; n = 6). The precise technique is described in Additional file 1. The results, calculated from a standard EBD curve (0.5–25 mg/l), are expressed in µg of Evans blue dye/g of dry tissue.

The selective B1R antagonist, R-954, was injected subcutaneously at a dose of 200 µg/kg prior to CLP and 24 h after surgery as a “prophylactic” treatment regimen (donation by R. Couture, see details in Additional file 1) [12].

Mean arterial pressure (MAP) was measured before surgery and before sacrifice 30 h after surgery. We anesthetized the mice in preparation for surgery. Femoral arterial pressure was monitored after a stabilization period of 5 min after anesthesia and every 30 s for 10 min using a blood pressure analyzer (Statham P10 EZ transducer coupled to a TA 4000; Gould, Eichstetten, Germany). Before the EBD challenge, the same protocol was used to measure the MAP on the contralateral femoral artery. The results that were published are related to the mean MAP value.

The renal expression of genes involved in inflammation (TNF-α, IL-1β and IL-6), endothelial function (eNOS, iNOS, kinin B2 receptor, VEGF receptor 1 and 2) and VE-cadherin was determined 30 h after the initial surgery. The fragments of kidneys were stored at − 80 °C in a stabilizing solution (RNAlater, Qiagen, Hilden, Germany) until RNA extraction. The precise techniques of mRNA extraction and reverse-transcriptase PCR are described in Additional file 1. All samples were run in duplicate, and results analyzed using SDS 2.3 software (Applied Biosystems, Thermo Fisher Scientific Inc., Waltham, Massachusetts). Relative mRNA expression levels were calculated with the delta–delta CT method and were normalized to GAPDH.

#### Second experimental series: effect of B1R pharmacological blockade on VE-cadherin expression

We studied the role of sepsis in a potential VE-cadherin destabilization in an in vivo model, and the impact of a B1R antagonist on inflammatory parameters, organ dysfunctions and VE-cadherin maintenance in two organs of interest: lungs and kidneys (WT sham n = 7; WT CLP n = 11; WT CLP R-954 n = 10).

#### Third experimental series: effect of B1R pharmacological blockade on survival

We studied survival following CLP for WT animals (n = 17) and WT animals that received R-954 as a “prophylactic” regimen (a subcutaneous injection of 200 µg/kg, prior to CLP and 24 h after surgery, n = 9), or as a “curative” regimen (injection of 100 µg/kg at H6, H24 and H48 post-surgery, n = 9).

### Biological samples

The blood samples were collected 30 h after surgery, under anesthesia, by cardiac puncture [13]. The serum samples obtained after centrifugation were immediately frozen at − 20 °C before being analyzed at the phenotyping department of the Toulouse Anexplo platform.

The biochemical determinations (ALP, ALT, AST, serum creatinine, serum lactates, and BUN) were carried out with the ABX Pentra 400 analyzer (Horiba ABX, Montpellier, France). Assessment of serum cytokine concentrations of IL-6 and IL-10 was performed using the Luminex technique (LXAMSM-02 2-plex Mouse Luminex Magnetic Assay, IL6, IL10, Bio-Techne, Abingdon, United Kingdom).

#### Histology and immunostaining

The kidneys and lungs were harvested and fixed in formalin 10%, dehydrated, and embedded in paraffin. Five-micrometer sections were stained with hematoxylin–eosin or used for immunohistochemistry.

We performed a semi-quantitative blind histological analysis for kidneys and lungs after hematoxylin–eosin staining. We quantified acute tubular necrosis lesions by the “Kidney Injury Score” and acute pulmonary lesions by the “Acute Lung Injury (ALI) score” described in Additional file 1 [14–16].

The antibodies used are described in Additional file 1. Confocal images were taken with the Confocal ZEISS LSM 510 microscope (Carl Zeiss, Jena, Germany). Immunohistochemical staining and fluorescence intensity were quantified using the FIJI distribution of the ImageJ software (version 1.51w, Wayne Rasband, National Institutes of Health, USA) [17].

### Statistical analysis

Inter-group comparisons for the unmatched samples were performed using the Kruskal–Wallis non-parametric test, followed by a Dunn’s test for the correction of risk inflation in multiple comparisons. For matched samples, we used the non-parametric Wilcoxon test. The log rank test was used to compare the survival rates between groups, followed by a Bonferroni correction for multiple comparisons. The mean survival times were evaluated with the Kaplan–Meier method. The analysis was performed with the GraphPad Prism software, version 6.01 for Windows (GraphPad Software, San Diego California USA). A value of p < 0.05 was considered as statistically significant. The results are expressed in median and interquartile range (IQR).

## Results

### The role of B1R in sepsis-induced vascular permeability

**Fig. 1:**
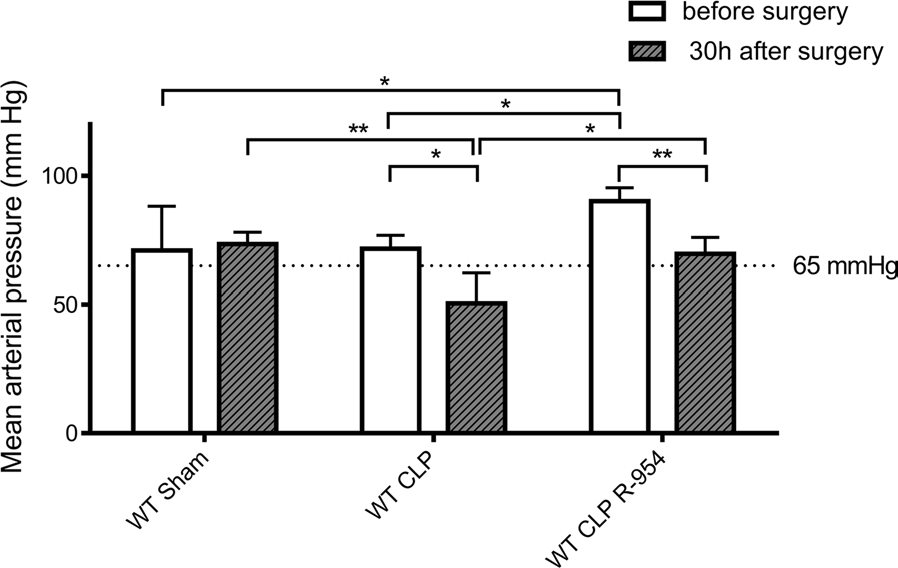
Mean arterial pressure of the different groups before and 30 h after surgery (in mmHg). Mean arterial pressure of the different groups before and 30 h after surgery (in mmHg): Non-parametric Wilcoxon signed-rank test for the comparison of paired groups before and 30 h after surgery; Kruskal–Wallis test followed by a Dunn’s correction for the other comparisons. WT sham: sham-operated C57BL/6J controls; WT CLP: CLP-induced septic C57BL/6J mice; WT CLP R-954: CLP-induced septic C57BL/6J mice that received prophylactic B1R antagonist R-954. n = 6 to 8 per group. *p < 0.05; **p < 0.01; ***p < 0.001. Results expressed in median ± IQR

MAP decreased 30 h after surgery in both groups who underwent CLP (Fig. 1, p < 0.05). Before surgery, the B1R blockade increased MAP (Fig. 1, p < 0.05). In the WT CLP R-954 group, CLP resulted in a lower significant decrease in MAP (Fig. 1, MAP at 30 h post-surgery (mmHg); WT CLP: 50.5 ± 26; WT CLP R-954: 69.7 ± 12.1. p < 0.05 between WT CLP vs WT CLP R-954).

**Fig. 2:**
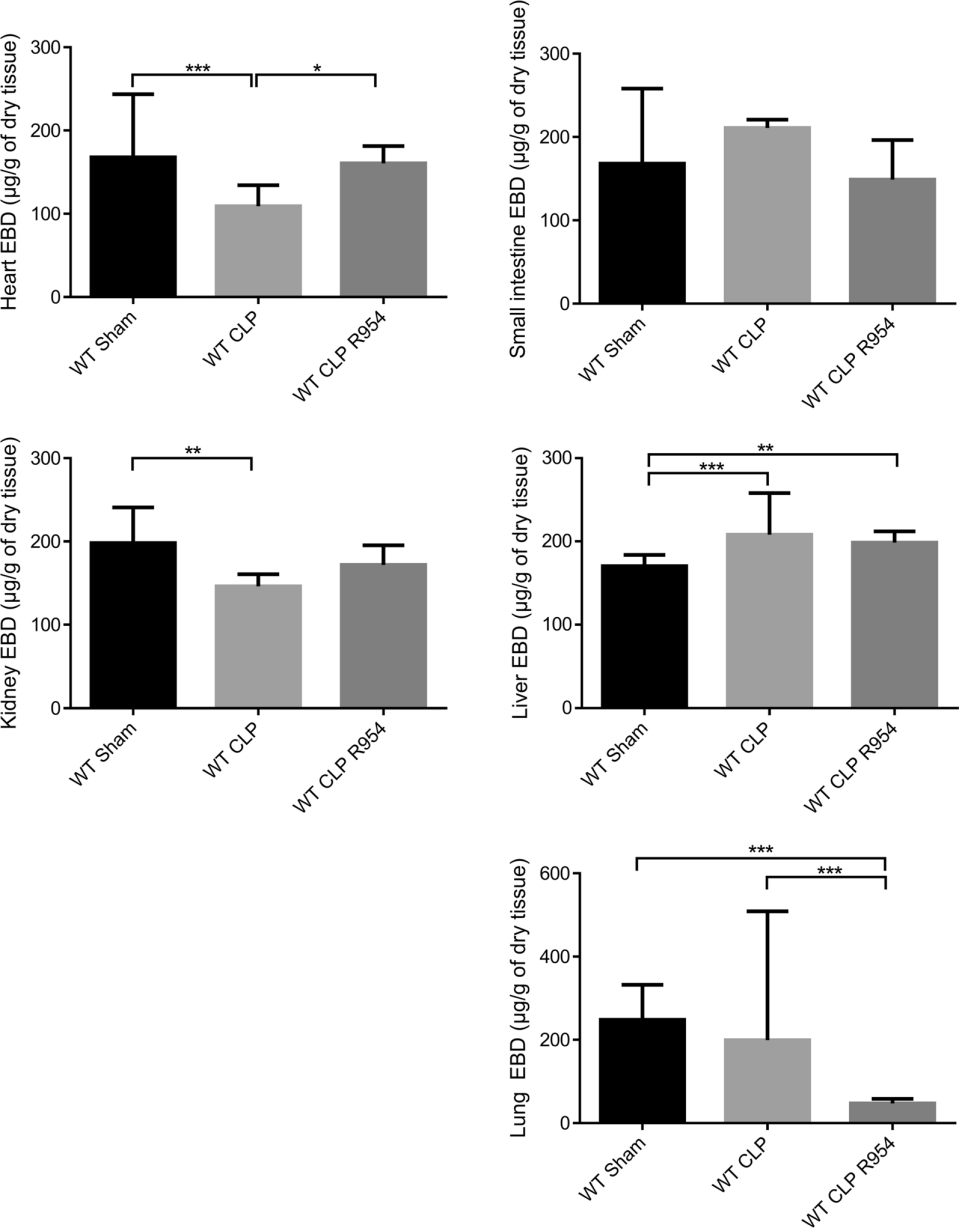
Evans blue dye vascular permeability assay (in µg/g of dry tissue). Evans blue dye vascular permeability assay (in µg/g of dry tissue): Kruskal–Wallis test followed by a Dunn’s correction. WT sham: sham-operated C57BL/6J controls; WT CLP: CLP-induced septic C57BL/6J mice; WT CLP R-954: CLP-induced septic C57BL/6J mice that received prophylactic B1R antagonist R-954. n = 6 to 8 per group. *p < 0.05; **p < 0.01; ***p < 0.001. Results expressed in median ± IQR

The organs presented two different permeability profiles. For autoregulated organs (heart, kidneys), a decrease in permeability was noted for the WT CLP group. The administration of a B1R antagonist prior to the induction of sepsis prevented a decrease in permeability (Fig. 2). On the other hand, we noted for the WT CLP group an increase in permeability for liver and a trend for an increased permeability for lungs during sepsis, an effect which is reduced by R-954 (Fig. 2). The lung benefited the most in terms of the effect of R-954 (Fig. 2, p < 0.05 between WT CLP vs WT CLP R-954).

### Renal expression of genes associated with inflammation, the function and the junctions of endothelial cells

**Fig. 3:**
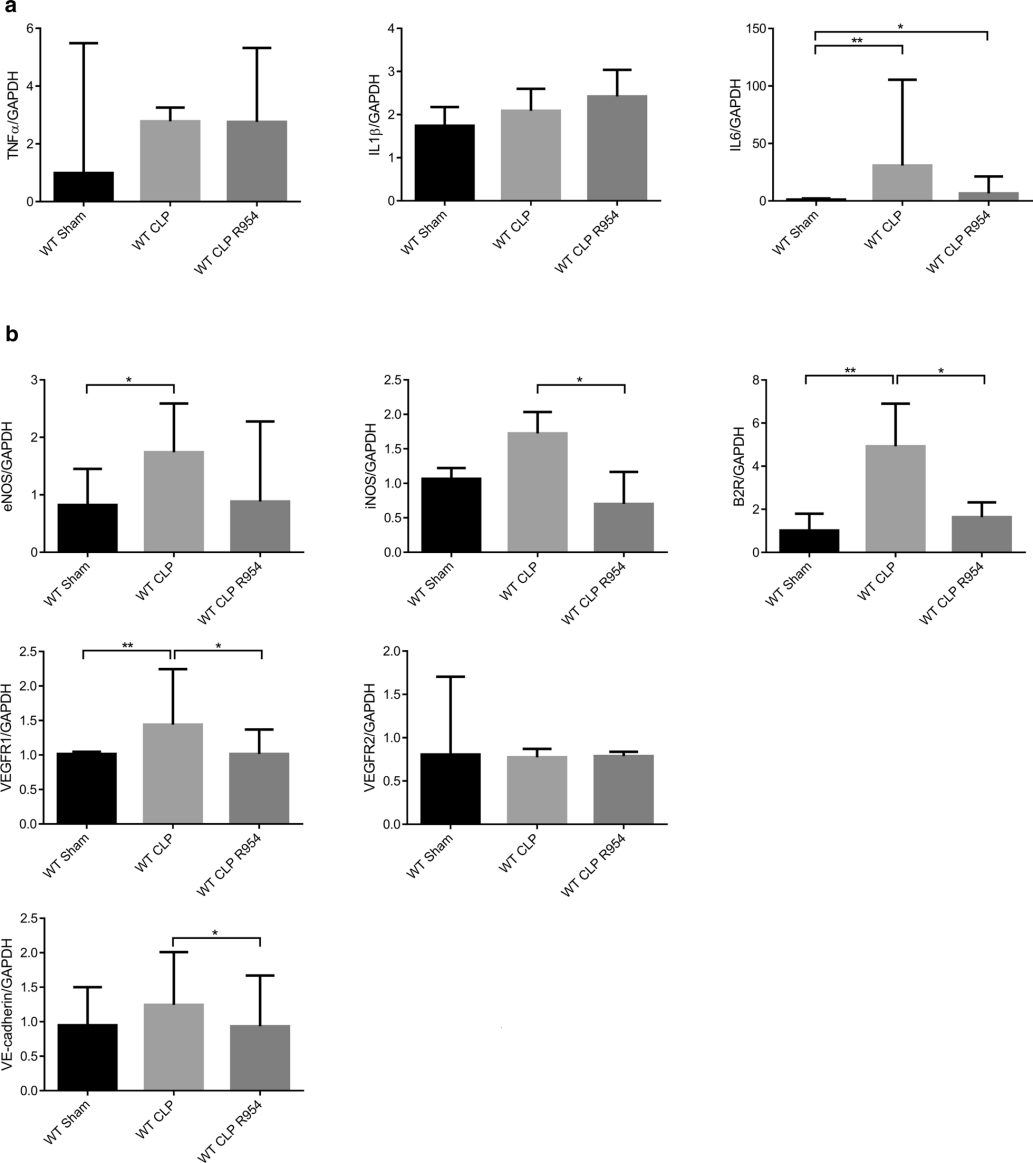
Renal expression of pro-inflammatory cytokines, molecules that reflect endothelial activation and VE-cadherin. **a** Rt-PCR of pro-inflammatory cytokines, TNF-α, IL-1β and IL-6, normalized by GAPDH. **b** Rt-PCR of eNOS, iNOS, kinin B2 receptor, VEGFR1, VEGFR2 and VE-cadherin normalized by GAPDH. WT sham: sham-operated C57BL/6J controls; WT CLP: CLP-induced septic C57BL/6J mice; WT CLP R-954: CLP-induced septic C57BL/6J mice receiving prophylactic B1R antagonist R-954. Kruskal–Wallis test followed by a Dunn’s correction. *p < 0.05; **p < 0.01. Results expressed in median ± IQR

For the kidney, only the IL-6 gene presented a significant increase in the CLP groups compared to shams (Fig. 3a).

The WT CLP group expressed more iNOS, B2R, VEGFR1 and VE-cadherin mRNAs than the WT CLP R-954 mice (Fig. 3b, p < 0.05). The eNOS expression was increased in the WT CLP group compared to the sham group (Fig. 3b).

### B1R blockade prevents inflammation and organ dysfunctions in septic mice

**Fig. 4:**
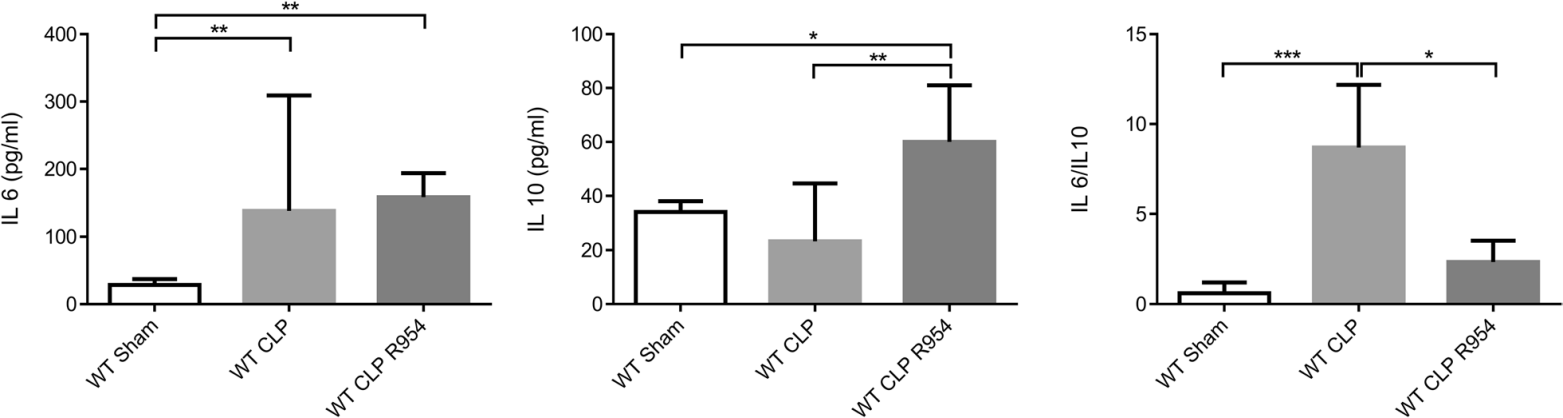
The role of R1B blockade in the inflammatory profile during sepsis. Determination of the serum concentration of the pro-inflammatory cytokine, IL-6, the anti-inflammatory cytokine, IL-10 and the IL-6/IL-10 ratio at H30 post-surgery in wild-type mice. n = 7 to 11 per group. WT sham: sham-operated C57BL/6J controls; WT CLP: CLP-induced septic C57BL/6J mice; WT CLP R-954: CLP-induced septic C57BL/6J mice that received prophylactic B1R antagonist R-954. Kruskal–Wallis test followed by a Dunn’s correction. *p < 0.05; **p < 0.01; ***p < 0.001. Results expressed in median ± IQR

At H30, the WT CLP R-954 group presented a more favorable anti-inflammatory balance than the WT CLP group with a higher IL-10 and a significantly lower IL-6/IL-10 ratio (Fig. 4).

**Fig. 5:**
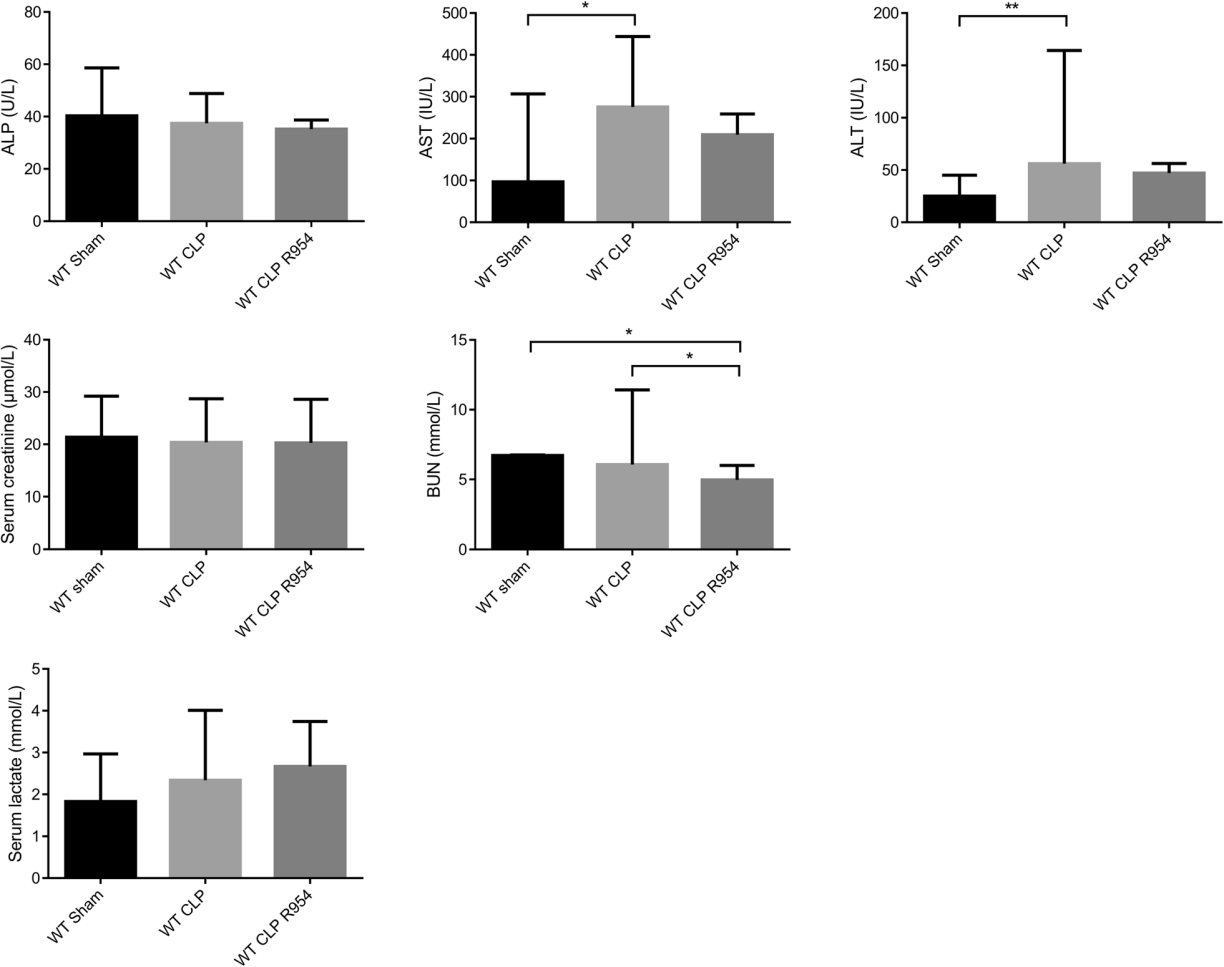
The role of R1B blockade on biological markers of organ dysfunction. Determination of the serum concentration of organ dysfunction markers: liver (ALP, ASAT, ALAT), kidneys (creatinine, urea) and lactatemia, at H30 post-surgery in wild-type mice. n = 7 to 10 per group. WT sham: sham-operated C57BL/6J controls; WT CLP: CLP-induced septic C57BL/6J mice; WT CLP R-954: CLP-induced septic C57BL/6J mice receiving prophylactic B1R antagonist R-954. Kruskal–Wallis test followed by a Dunn’s correction. *p < 0.05; **p < 0.01; ***p < 0.001. Results expressed in median ± IQR

**Fig. 6:**
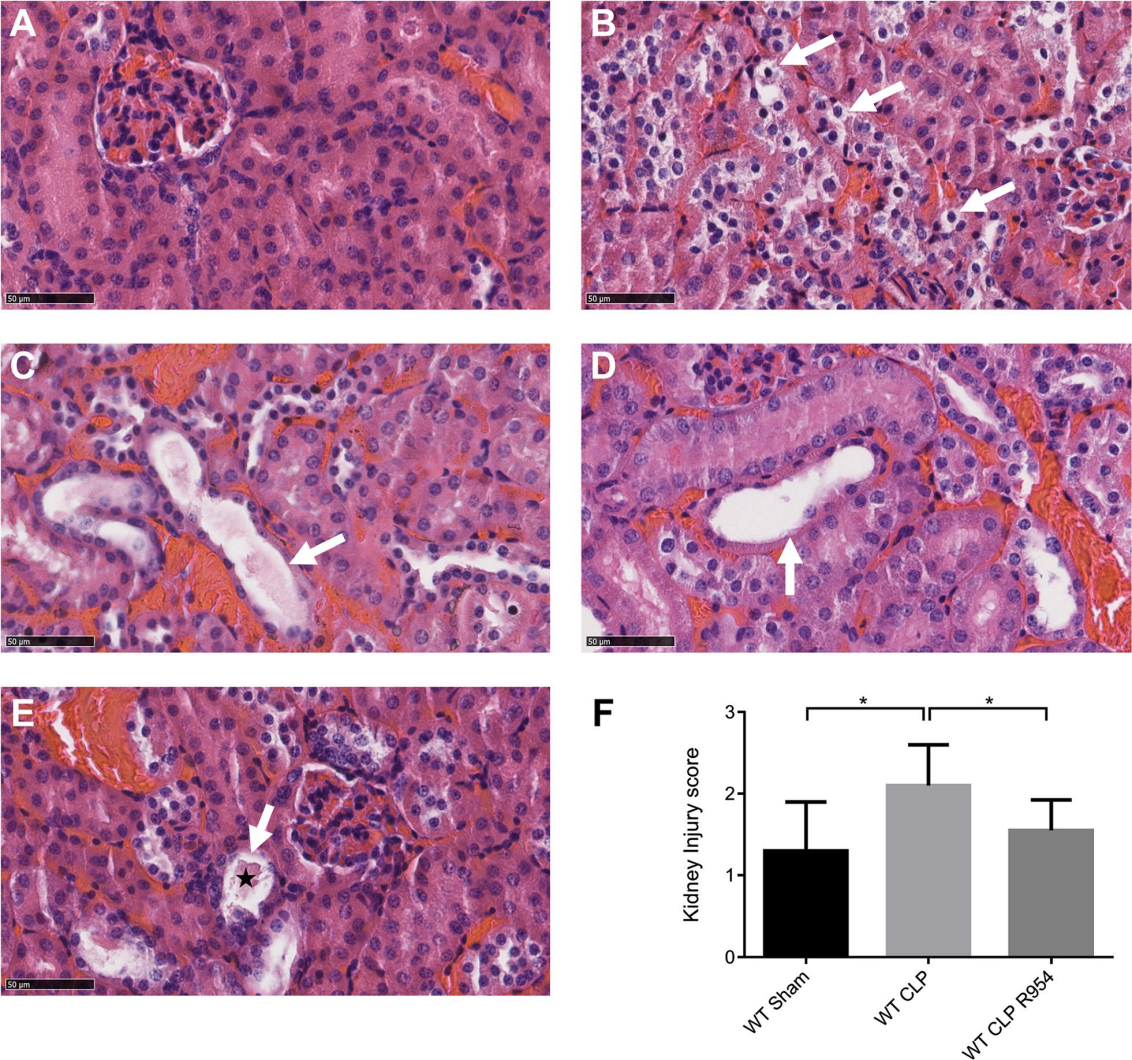
The impact of R1B blockade on renal histological lesions due to sepsis. Histological analysis of the renal cortex 30 h after sham surgery, CLP, or CLP with R-954 antagonist. Hematoxylin–eosin staining. Magnification ×400. Scale: 50 µm. **a** Normal renal parenchyma. **b** Renal tubular cell vacuolization lesion (white arrow). **c** Intratubular cast (white arrow). **d** Desquamation lesion (white arrow). **e** Example combining an intratubular cast (black star) and desquamation lesion (white arrow). **f** Mean Kidney Injury Score for 10 fields per slide evaluated in a blinded fashion (optical microscopy, ×400 magnification). WT sham: sham-operated C57BL/6J controls; WT CLP: CLP-induced septic C57BL/6J mice; WT CLP R-954: CLP-induced septic C57BL/6J mice receiving prophylactic B1R antagonist R-954. Kruskal–Wallis test followed by a Dunn’s correction. n = 7 to 11 per group. *p < 0.05. Results expressed in median ± IQR

For the CLP group, we noted a significant elevation in transaminases (Fig. 5). For this group, we observed no increase in serum creatinine, while the histology confirmed the presence of significant lesions, with a Kidney Injury Score of 2.1 ± 0.8 *vs*. WT CLP R-954 with a score of 1.55 ± 1.15 and *vs.* shams with a score of 1.3 ± 1 (Figs. 5 and 6).

**Fig. 7:**
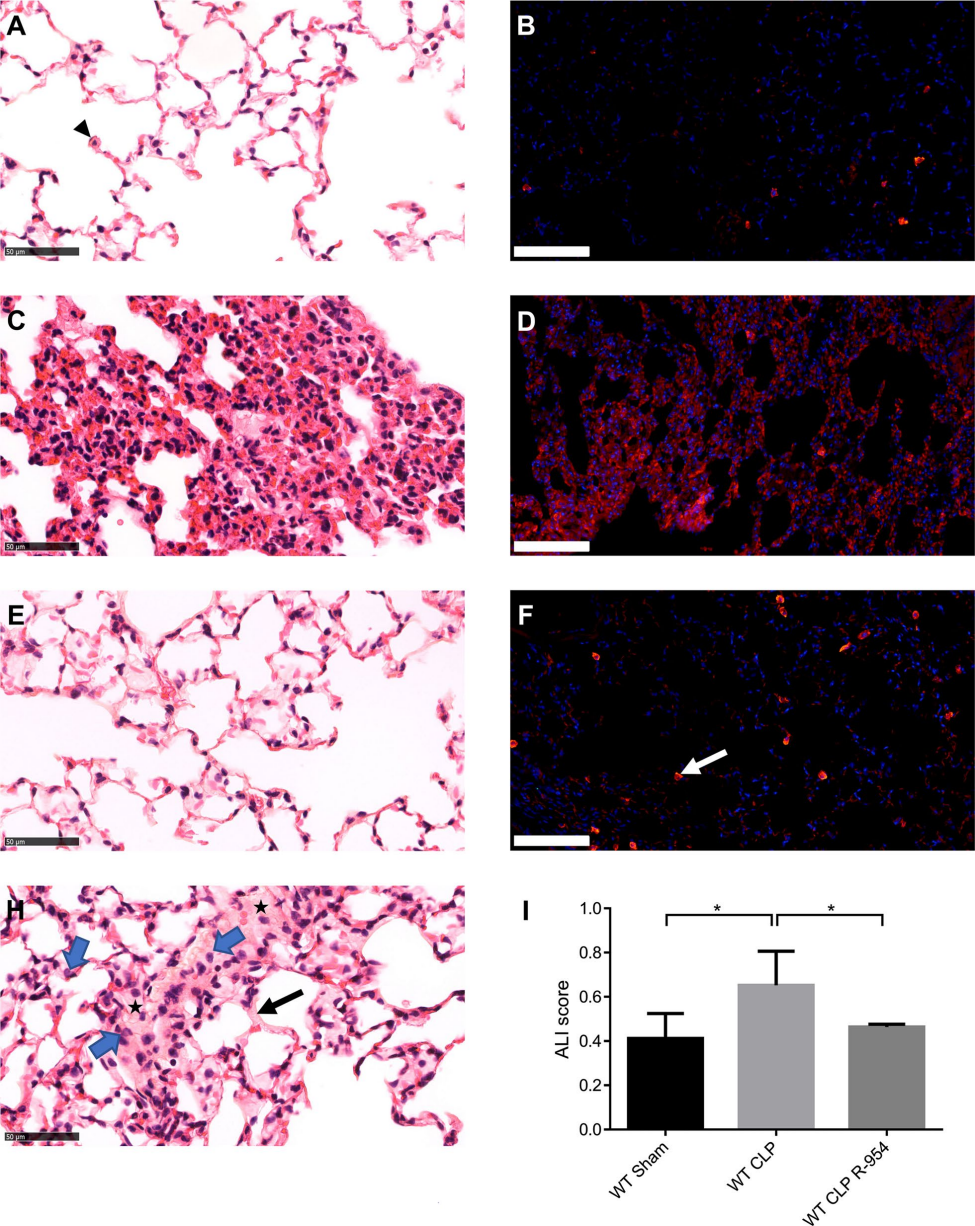
The impact of R1B blockade on pulmonary histological lesions due to sepsis. Pulmonary histological analysis 30 h after sham surgery, WT CLP, or WT CLP that received R-954 antagonist. **a**, **c**, **e**, **h** Hematoxylin–eosin staining. Magnification ×400. Scale: 50 µm. **b**, **d**, **e** Immunofluorescence of CD45 positive cells (panleukocyte marker) in red. Nuclei are counterstained with DAPI (blue). Magnification ×200. Scale: 100 µm. **a** Pulmonary parenchyma of a sham mouse. The head of the arrow indicates an alveolar macrophage. **b** Infiltration of CD45 + cells in a sham mouse parenchyma. **c** WT CLP mouse parenchyma with acute histological pulmonary lesions characterized by alveolar septa thickening, polynuclear neutrophil infiltration and fibrin strands. **d** Infiltration of CD45 + cells in the parenchyma of a WT CLP mouse. **e** Pulmonary parenchyma of a WT CLP R-954 mouse. **f** Infiltration of CD45 + cells in the parenchyma of a WT CLP R-954 mouse. The white arrow indicates an alveolar macrophage. **g** Histological lesions of an acute lung injury: polynuclear neutrophils (blue arrows), intra-alveolar fibrin strands (stars), hyaline membrane (black arrow). **h** Mean Acute Lung Injury score for 5 fields per slide evaluated in a blind (optical microscopy, magnification ×400). WT sham: sham-operated C57BL/6J controls; WT CLP: CLP-induced septic C57BL/6J mice; WT CLP R-954: CLP-induced septic C57BL/6J mice that received prophylactic B1R antagonist R-954. Kruskal–Wallis test followed by a Dunn’s correction. n = 7 to 11 per group. *p < 0.05. Results expressed in median ± IQR

Similarly, the mice that received R-954 had fewer acute pulmonary lesions at H30 post-surgery (Fig. 7e). Their ALI score was lower than in the WT CLP mice (WT CLP R-954: 0.46 ± 0.14 vs. WT CLP: 0.65 ± 0.32, p < 0.05, Fig. 7i), and about the same as that of the sham group (WT sham: 0.41 ± 0.18, Fig. 7i).

### B1R blockade stabilizes VE-cadherin during sepsis

**Fig. 8:**
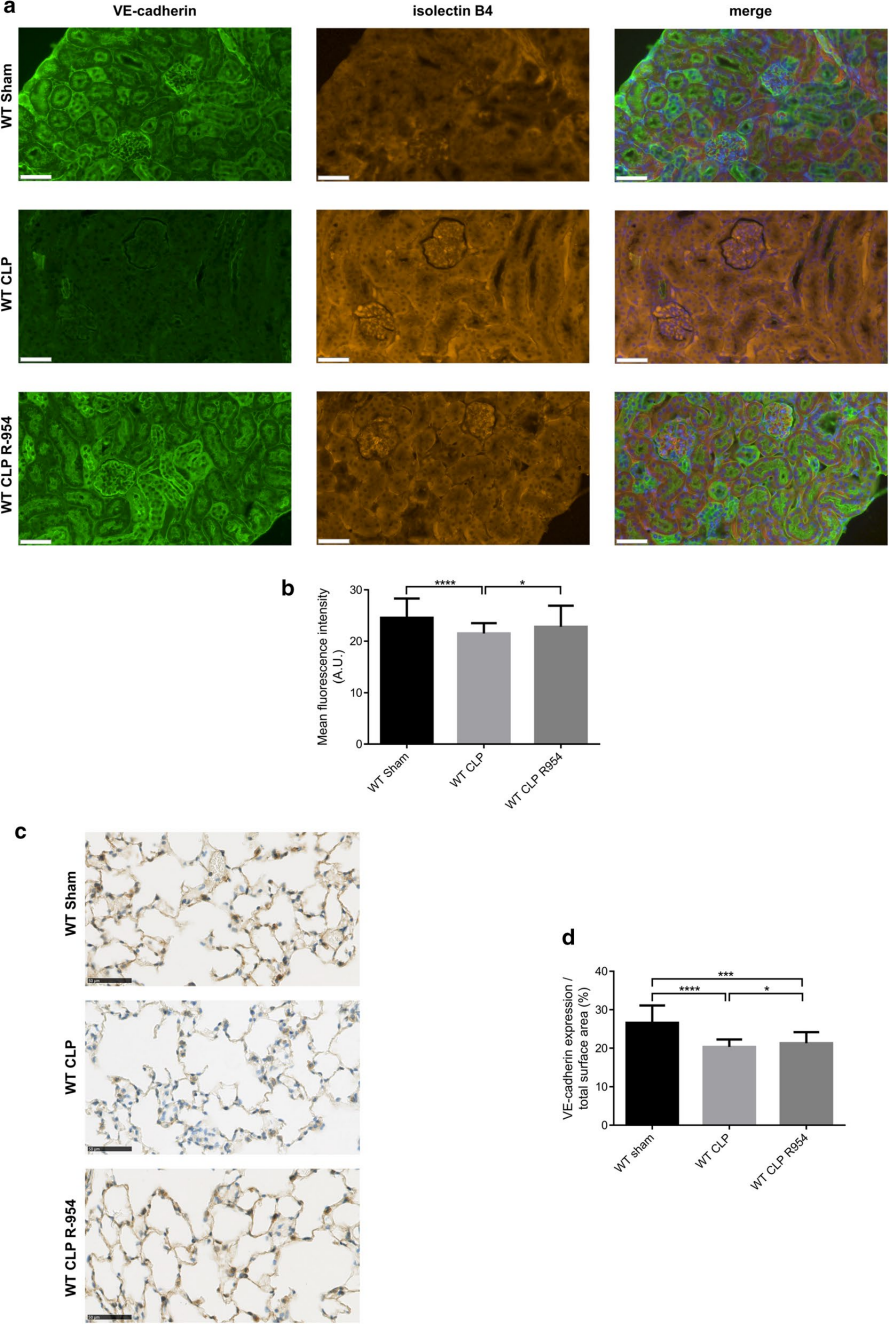
VE-cadherin expression in the renal cortex and lungs. **a** VE-cadherin immunostaining in the renal cortex of the WT sham, WT CLP and WT CLP R-954 groups. Specific fluorescence: green for VE-cadherin, orange for isolectin B4 (endothelial cell marker), blue for cell nuclei (DAPI). Scale: 50 µm. **b** Mean fluorescence intensity established for 10 fields selected randomly by slide, magnification ×400. A.U.: arbitrary unit; n = 7 to 11 per group. **c** VE-cadherin immunostaining in the lungs of the WT sham, WT CLP and WT CLP R-954 groups. Scale: 50 µm (optical microscope magnification ×400). **d** VE-cadherin expression in relation to the total immunostained surface area, established for 10 fields selected randomly by slide, with magnification ×400 (in %); n = 7 to 10 per group. WT sham: sham-operated C57BL/6J controls; WT CLP: CLP-induced septic C57BL/6J mice; WT CLP R-954: CLP-induced septic C57BL/6J mice that received prophylactic B1R antagonist R-954. Kruskal–Wallis test followed by a Dunn’s correction *p < 0.05; ***p < 0.001; ****p < 0.0001. Results expressed in median ± IQR

For the renal cortex, VE-cadherin was significantly decreased by sepsis (mean fluorescence intensity (MFI), in the arbitrary unit (A.U.): 21.5 ± 3.4 for WT CLP vs. 24.6 ± 6.6 for WT sham p < 0.0001). R-954 partially prevented this destabilization (22.8 A.U. ± 6.3 for WT CLP R-954, p non-significant *vs.* WT sham and p < 0.05 vs. WT CLP) (Fig. 8a, b).

In the lungs, VE-cadherin expression was reduced for WT CLP mice in comparison to the shams (% median expression/total surface area was respectively 20.4% ± 7.1 vs. 26.6% ± 9.1, p < 0.0001). B1R antagonizing enabled partial restoration of VE-cadherin in comparison to the WT CLP group (21.3% ± 5.3 for WT CLP R-954; p < 0.05 vs. WT CLP and p < 0.001 vs. WT sham) (Fig. 8c, d).

### B1R antagonizing limits p-eNOSser1177 expression in the lungs during sepsis

**Fig. 9:**
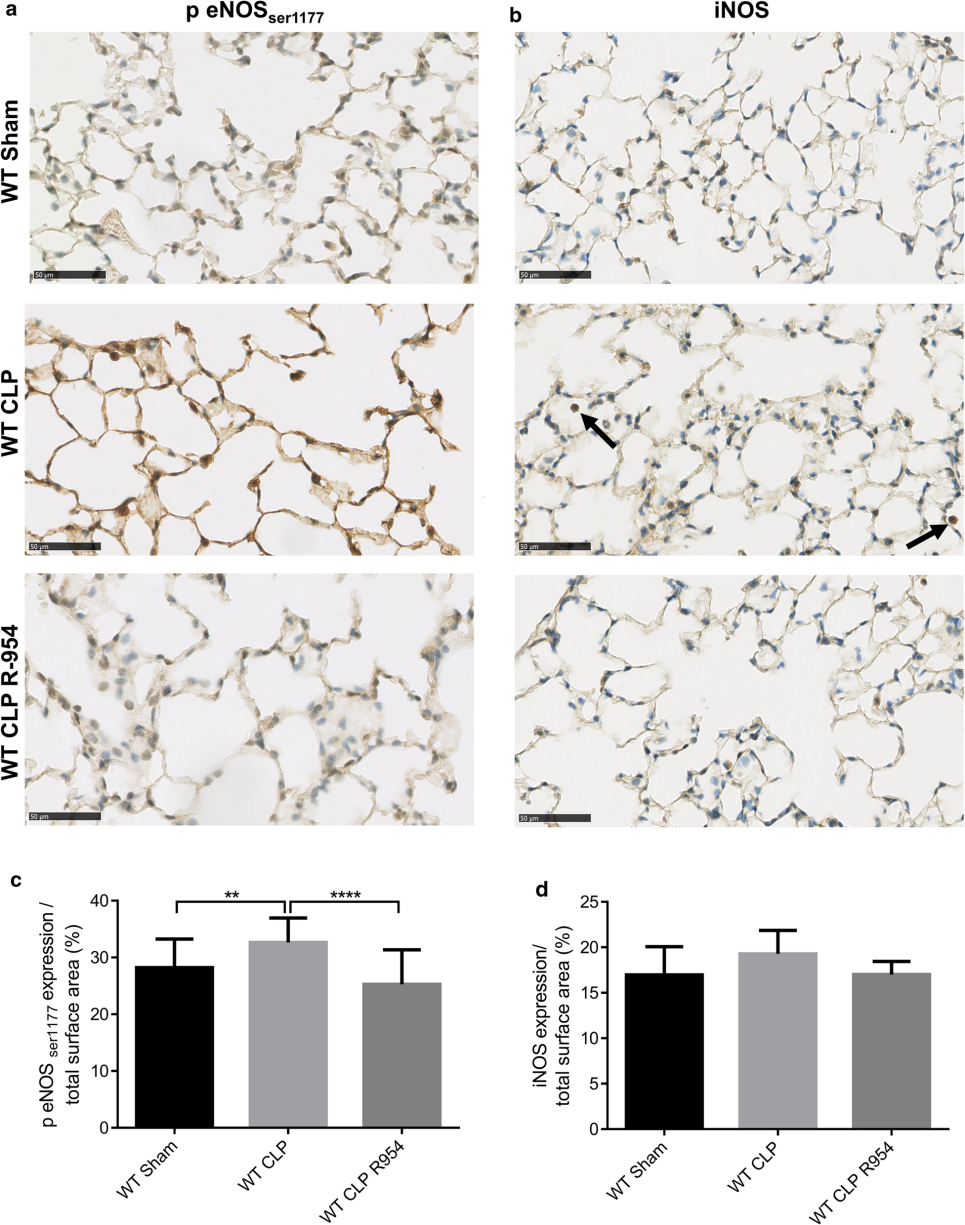
NO synthases expression in the lungs of WT sham, WT CLP and WT CLP R-954 mice. **a** p-eNOS_ser1177_ immunostaining in the lungs of the WT sham, WT CLP and WT CLP R-954 groups. Scale: 50 µm (optical microscope magnification ×400). **b** iNOS immunostaining in the lungs of the WT sham, WT CLP and WT CLP R-954 groups. The black arrows indicate intra-alveolar macrophages marked by iNOS. Scale: 50 µm (optical microscope magnification ×400). **c** p-eNOS_ser1177_ expression in relation to the immunostained total surface area, established for 10 fields selected randomly by slide, with magnification ×400 (in %). (D) iNOS expression in relation to the immunostained total surface area, established for 10 fields selected randomly by slide, with magnification ×400 (in %). WT sham: sham-operated C57BL/6J controls; WT CLP: CLP-induced septic C57BL/6J mice; WT CLP R-954: CLP-induced septic C57BL/6J mice receiving prophylactic B1R antagonist R-954. For **c** and **d** Kruskal–Wallis test followed by a Dunn’s correction. n = 7 to 10 per group. **p < 0.01; ****p < 0.0001. Results expressed in median ± IQR

We paid particular attention to the expression of iNOS and p-eNOS_ser1177_ isoforms in the lungs. While we found no difference in the iNOS expression at H30, the WT CLP group showed a higher level of p-eNOS (% median expression/total surface area of 32.6% ± 8.2 for WT CLP, compared to 28.2% ± 7.5 in the shams and 25.3% ± 8.4 in WT CLP R-954) (Fig. 9).

### B1R blockade improves the survival of septic animals

**Fig. 10:**
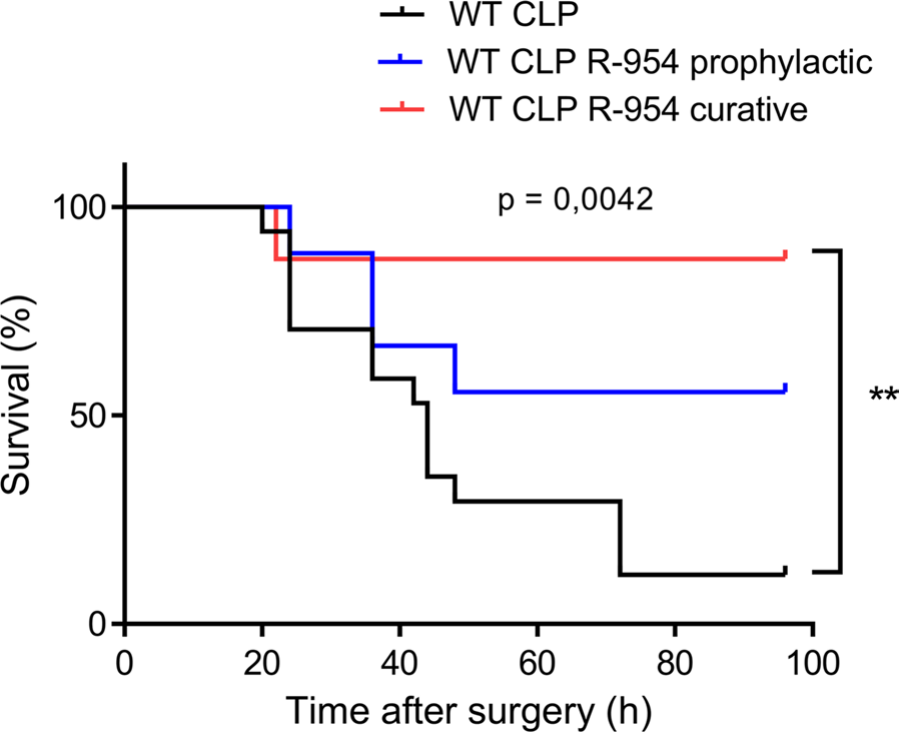
The influence of R1B pharmacological blockade on the survival of septic mice. Survival following CLP for WT animals (n = 17) and WT animals that received R-954 in a prophylactic regimen (n = 9), or in a curative regimen (n = 9). Log rank test followed by Bonferroni correction for multiple comparisons **p < 0.01

The survival analysis showed significant differences between the groups (p < 0.005), with a median survival of 44 h for the WT CLP group. Median survival for groups that received R-954 as a “prophylaxis” or a “cure” could not be determined considering that the number of deaths was low at the end of the observation period of 96 h (56% survival for the prophylactic group and 87.5% for the curative group). Only 11.8% of the mice in the WT CLP group survived at the end of the observation period. Due to the number of animals, the analysis only showed a significant difference between the WT CLP and the WT CLP R-954 curative groups (Fig. 10, p < 0.01).

## Discussion

The aim of our study was to determine the involvement of B1R in sepsis-induced vascular hyperpermeability beyond the first 24 h of progression. To our knowledge, our study is the first to establish a connection between VE-cadherin stabilization (transmembrane component of the adherens junctions which regulate endothelial integrity), the B1R pharmacological blockade and a reduction in vascular hyperpermeability for two sepsis target organs [18, 19]. Endothelial dysfunction was reduced, which improved the organ dysfunctions and the survival of animals that received R-954. This was consistent with two recent studies that used a B1R antagonist, BI113823, administered orally. This molecule attenuated lung injuries secondary to LPS or CLP [20, 21]. In another model of acute lung injury caused by lipopolysaccharide inhalation, R-954 counteracts the role of B1R and prevented the airway hyperreactivity, the increased cellular infiltration and protein content in the bronchoalveolar lavage fluid, and inhibited the expression of cytokines/chemokines [22]. As was the case in these studies, we found the inflammatory profile for WT CLP R-954 mice to be less severe than for the other septic group. In fact, B1R induced NF-κB and therefore the production of TNF-α and IL-1β, as well as CXCL5, an important mediator of neutrophil recruitment [4, 5, 22–25]. We use in our study low dose of buprenorphine (0.05 mg/kg every 12 h) to achieve analgesia as stated by the Office of Laboratory Animal Welfare [26]. Morphine is known to increase proinflammatory mediators, however low dose of buprenorphine seems to have no effect on mortality and inflammatory response [27, 28]. Moreover, we gave this analgesic drug to all experimental groups, hence we believe that buprenorphine is not a cofounding factor in our model. The same goes for ketamine and fluid resuscitation.

In inflammatory conditions, interendothelial cell clefts open up [29–31]. This inflammatory status was present in the WT CLP mice. 30 h after the beginning of sepsis, they had a serum cytokine balance that was favorable to inflammation and an increase in renal IL-6 transcription. While at first glance the permeability profile was different in the WT CLP group, our in vivo results concerning VE-cadherin expression confirmed AJ destabilization in two organs subjected to septic dysfunctions: the kidney (autoregulated organ) and the lung (non-autoregulated). Renal expression of the VE-cadherin gene increased in the WT CLP group while protein expression decreased. In fact, vascular permeability controlled by VE-cadherin was regulated in many ways, notably by VE-cadherin cleavage or internalization [32]. This opposite effect on mRNA and protein expression has been observed before in mice kidneys after LPS challenge, probably to compensate for loss of the protein function [33, 34].

These results should be taken into account considering the physical determinants of capillary filtration, described by the revised Starling principle [30, 35]:

$${\text{Jv}} = {\text{Kf}}\left[ {\left( {{\text{Pc}} - {\text{Pi}}} \right) -\upsigma\left( {\Pi {\text{c}} - \Pi {\text{g}}} \right)} \right]$$

(Jv: fluid filtration rate, Kf: filtration coefficient, Pc: capillary hydrostatic pressure, Pi: interstitial hydrostatic pressure, σ: reflection coefficient, Пc: capillary oncotic pressure, Пg: sub-glycocalyx oncotic pressure).

The revised law proposes that the glycocalyx acts as the effective osmotic barrier. The glycocalyx is a dynamic structure which is directly impacted by the composition of plasma and exogenous fluids. For example, albumin is particularly necessary to maintain normal myocardial microvascular permeability [36]. In kidney, fluid absorption is sustained by local epithelial secretions, which flush interstitial plasma proteins into the lymphatic system [30].

The WT CLP mice had a MAP of 50 mm Hg which implies a decrease in capillary hydrostatic pressure (Pc). For autoregulated organs (heart, kidney), the decrease in fluid filtration rate was pronounced because the MAP was below the autoregulation threshold. For the other organs, the increase in permeability outweighed the decrease in Pc. On the contrary, an increase in MAP restored capillary hydrostatic pressure and consequently the filtration force.

The B1R blockade induces a different hemodynamic and permeability profile. It improves organ dysfunction by maintaining the organ perfusion pressure (MAP) and by stabilizing the interendothelial junctions. WT CLP R-954 mice had higher MAP than WT CLP mice. As a result, R-954 restored Pc in the heart and kidney and finally contributed to Evans Blue extravasation. A higher MAP was noted before surgery for the WT CLP R-954 group than for the others, which might correspond to an effect solely attributable to the antagonist [37]. Even if significant, the fall in MAP in this group before CLP and at 30 h may not be linked to sepsis, but to initial hypertension. The other organs showed a decrease in permeability in the R-954 group as reported in the literature [21, 38, 39]. Moreover, the significant decrease in extravasation of EBD in the lungs is partially explained by the action of B1R in the pulmonary vascular bed. B1 receptors have been shown to mediate strong contractile response in pulmonary arteries and likely participate in the increase of pulmonary vascular resistance [40, 41]. Inhibition of B1R attenuates this phenomenon [40]. In general, it would seem that B1R activation consistently produces vasoconstriction in veins and vasodilatation in arteries [42]. Studies suggest that central B1R may play a role in the pathology of essential hypertension. However normal regulation of arterial pressure does not appear to involve this receptor since B1R KO mice display a normal phenotype [43, 44].

VE-cadherin is the target of a plethora of signaling pathways, which can lead to a break in the endothelial barrier. We paid particular attention to the NO pathways, known to induce vascular hyperpermeability, especially since kinin receptors activate eNOS and iNOS. eNOS derived-NO is considered to be beneficial in the vessel wall because this enzyme produces appropriate amounts of NO, unlike the high NO output generated by iNOS [45–47]. We found no iNOS staining in the lung at a late phase after surgery, but a p-eNOS_ser1177_ overexpression in WT CLP mice in comparison to the group that received the antagonist. This form is known to lead to VE-cadherin destabilization by enabling phosphorylation and S-nitrosylation of this cadherin and its partner proteins [18, 48–50]. Moreover, in an inflammatory endothelium, eNOS is capable of producing amounts of NO that are identical to iNOS for extended periods [47, 51].

In a similar manner to the different permeability patterns, the mice with blocked B1R did not have the same NOS expression profile as the WT mice. What was surprising at first glance was the difference in eNOS expression between the two septic groups, WT CLP and WT CLP R-954, whether it concerned the renal transcription or the activated protein form in the lung. Actually, B2R is reputed to be an eNOS activator, but B1R is also capable of similar effects because these two receptors share the same signaling pathways [4–6]. Various researches have shown either negative regulation of eNOS transcription in the absence of B1R, a co-localization of B1R and eNOS, or new potential transduction pathways that enable the activation of eNOS by B1R [52–55]. In addition, B1R is capable of activating the PI3/Akt pathway, which phosphorylates eNOS in serine 1177 [56–58].

B1R blockade improved the survival of septic animals only in the group receiving treatment after induction of sepsis. Mice receiving treatment before CLP, however, seem to survive better than septic mice without any treatment. The lack of significance can be explained by an insufficient number of animals in this experimental group. The more favorable profile of survival in the group receiving R-954 after CLP emphasizes the Janus-faced aspect of B1R. B1R enables the transmigration of neutrophils to inflammatory sites by causing the overexpression of the chemokine CXCL5 on the surface of endothelial cells [59]. Therefore, blocking it immediately could limit an immune response favorable to the elimination of the pathogen. The following two notions can be derived from this result: it is continuous and deregulated stimulation of B1R that is harmful and a B1R antagonist might therefore have a place in the armamentarium against septic shock because the action also seems more favorable when the inflammatory response is already initiated.

## Conclusions

B1R blockade promotes the anti-inflammatory balance and improves the hemodynamics and the maintenance of VE-cadherin by limiting the activation of eNOS. This pharmacological blockade has a greater protective effect after the induction of sepsis. The antagonist that we used, R-954, authorized for a phase 1 study in humans by Health Canada, could provide a promising new therapeutic strategy for septic shock management.

## Supplementary information

**Additional file 1.** Materials and methods.

## Data Availability

The datasets used and/or analysed during the current study are available from the corresponding author on reasonable request.

## Abbreviations

ALI: Acute lung injury
B1R: Kinin B1 receptor
B2R: Kinin B2 receptor
CLP: Cecal ligation and puncture
EBD: Evans blue dye
Jv: Fluid filtration rate
Kf: Filtration coefficient
LPS: Lipopolysaccharide
MAP: Mean arterial pressure
MFI: Mean fluorescence intensity
Pc: Capillary hydrostatic pressure
Pi: Interstitial hydrostatic pressure
VE-cadherin: Vascular endothelial cadherin
WT: Wild type
Пc: Capillary oncotic pressure
Пg: Sub-glycocalyx oncotic pressure
σ: Reflection coefficient
