# Supplementary material for: Kinin B1 receptor: a potential therapeutic target in sepsis-induced vascular hyperpermeability
Source: J Transl Med. 2020 Apr 19;18:174. doi: 10.1186/s12967-020-02342-8 (PMC7168845; doi:10.1186/s12967-020-02342-8)
Supplement: Supplementary file 1 — Additional file 1. Materials and methods. [file 12967_2020_2342_MOESM1_ESM.docx]

**Additional File 1: Material and Methods**

Flow charts of experimental series:

**Figure S1: Flow charts of the three experimental series**

WT sham: sham-operated C57BL/6J controls; WT CLP: CLP-induced septic C57BL/6J mice; WT CLP R-954: CLP-induced septic C57BL/6J mice receiving prophylactic B1R antagonist R-954.

Evans blue dye (EBD) protocol:

Briefly, the mice received a bolus of EBD (30 mg/kg) by an internal left jugular catheter 15 minutes after anesthesia. After 15 minutes of circulation of the EBD, the animals were sacrificed by exsanguination with an aortic catheter. The circulatory system was then rinsed with heparinized serum (20 IU/ml) administered by the same catheter. The organs of interest were removed, rinsed in physiological saline, dried on absorbent paper and weighed. Half of each tissue was desiccated at 60°C for 24 hours. The other half was placed in formamide, ground and left in a water bath for 24 hours at 24°C. Supernatant fluids were separated from tissues by centrifugation. The concentration of EBD in the supernatant fluids was determined by spectrophotometry at 620 nm (Beckman DU 640).

Selective kinin B1 receptor antagonist:

We used a selective kinin B1 receptor antagonist, R-954 (Ac-Orn-[Oic², α-MePhe5, D-βNal7, Ile8]desArg9-bradykinin) (kinin B1 receptor Ki = 2.4 nmol/l; kinin B2 receptor Ki > 10 µmol/l, donation by R. Couture, Department of Pharmacology and Physiology, University of Montreal, Montreal, QC, Canada.) [1]. R-954, dissolved in normal saline solution, was injected subcutaneously, at a dose of 200 µg/kg prior to cecal ligation and puncture (CLP) and 24 hours after surgery as prophylactic treatment. A curative regimen of R-954 was injected at a dose of 100 µg/kg at H6, H24 and H48 post-surgery.

RNA extraction and reverse transcriptase PCR techniques:

Total RNAs were extracted with an RNeasy Plus Mini Kit (Qiagen). RNA quality and integrity were verified by microfluidic capillary electrophoresis using an Experion Std Sens Kit (Bio-Rad, Hercules, California), and the concentration was measured by UV spectrophotometry on an ND-1000 (Nanodrop, Wilmington, Delaware). One µg of total RNA was introduced into a reaction of reverse transcriptase-PCR using Superscript II Reverse Transcriptase (Invitrogen Co, Carlsbad, California). Real-time PCR was carried out with 250 ng of cDNA on a Taqman 7900 device (Applied Biosystem, Foster City, California) adapted to the thermal cycler FastBy, with the use of SYBR^®^Green chemistry (DyNAmo Flash Sybr Green qPCR kit, Finnzymes, Thermo Fisher Scientific Inc., Waltham, Massachusetts). The following primer pairs (forward and reverse) were used to quantify mRNA expression levels: TNF-α, 5’-TCTGTCTACTGAACTTCGGG-3’ and 3’-TGATCTGAGTGTGAGGGTC-5’ ; IL1-β, 5’-GAGCACCTTCTTTTCCTTCATCTT-3’ and 3’-AACGTCACACACCAGCAGGTT-5’ ; IL6, 5’-AACTCTAATTCATATCTTCAACCAAG-3’ and 3’-TCCTTAGCCACTCCTTCTG-5’ ; eNOS, 5’-TTGTCTGCGGCGATGTCA-3’ and 3’-GAATTCTCTGCACGGTTTGCA-5’ ; iNOS, 5’-CAGCTGGGCTGTACAAACCTT-3’ and 3’-GGGATCTGAATGTGATGTTTGCT-5’ ; BK-RB2, 5’-TCTTTGTCCTCAGCGTGTTC-3’ and 3’-AGTTATTGGCGATGGTGATGG-5’ ; VEGF R1, 5’-CAATGTGGAGAGCCGAGACA-3’ and 3’-ACATGCACGGAGGTGTTGAA-5’ ; VEGF R2, 5’-ACTGCAGTGATTGCCATGTTCT-3’ and 3’-TCATTGGCCCGCTTAACG-5’ ; VE-cadherin, 5’-AGGTATTCAACGCATCTGTG-3’ and 3’-CGATTTGGTACAAGACAGTGG-5’; GAPDH, 5’-AGGTCGGTGTGAACGGATTTG-3’ and 3’-TGTAGACCATGTAGTTGAAGGTCA-5’.

Immunohistochemistry and immunofluorescence antibodies:

The preparation of the slides and standard hematoxylin-eosin staining were performed by the Toulouse Hospitals Cancer Biological Resources Center.

The following rabbit antibodies were used for immunostaining: mouse eNOS Ser1177 (1/50, SAB4300128, Sigma Aldrich), iNOS (1/100, PA3-030A, Thermo Fischer Scientific) and VE-cadherin (1/100, ab33168, ABCAM). Pan-leukocyte marking was done with a rat antibody, anti-CD45 (1/50, 550539, BD Pharmingen), coupled with a secondary antibody, Alexa fluor 488 goat anti-rat (A11006, Life Technologies). The antibody-antigen complexes were detected by a secondary antibody combined in a complex with an avidin-biotin-peroxidase system, through which diaminobenzidine is revealed. Nuclei were counterstained with hematoxylin. For VE-cadherin immunofluorescence, we used Alexa Fluor 488 conjugated secondary antibodies (A-01108, Thermo Fisher Scientific). The nuclei were counterstained with DAPI (4′,6′-diamidino-2-phenylindole), and the endothelial cells with Alexa Fluor 568 conjugated isolectin B4 (isolectin GS-IB_4,_ I21412, Thermo Fisher Scientific).

Immunohistochemical staining and fluorescence intensity were quantified using the FIJI distribution of the ImageJ software (version 1.51w, Wayne Rasband, National Institutes of Health, USA) [2]. The macros required were designed by Rémy Flores-Flores (TRI-GENOTOUL Cell Imagery Platform, I2MC).

Acute Kidney Injury (AKI) score:

We performed a renal histological analysis after hematoxylin-eosin staining to identify acute tubular necrosis lesions (cell vacuolization, intratubular cast and desquamation) quantified by a semi-quantitative blind evaluation, the "Kidney Injury Score": 0: no lesion; 1: 1-10%; 2: 11-25%; 3: 26-45%; 4: 46-75%; and 5: >76% [3, 4].

Acute lung injury (ALI) scoring system [5]:

| Parameter | Score per field | | |
| --- | --- | --- | --- |
|  | 0 | 1 | 2 |
| A. Neutrophils in the alveolar space | none | 1-5 | >5 |
| B. Neutrophils in the interstitial space | none | 1-5 | >5( |
| C. Hyaline membranes | none | 1 | >1 |
| D. Proteinaceous debris filling the airspaces | none | 1 | >1 |
| E. Alveolar septal thickening | < 2x | 2x-4x | >4x |

Score = [(20 x A) + (14 x B) + (7 x C) + (7 x D) 1 (2 x E)]/(number of fields x 100)

The ALI score is a continuous value between zero and one (inclusive).

References:

1. Neugebauer W, Blais PA, Halle S, Filteau C, Regoli D, Gobeil F, Jr.: **Kinin B1 receptor antagonists with multi-enzymatic resistance properties.** *Can J Physiol Pharmacol* 2002, **80:**287-292.

2. Schindelin J, Arganda-Carreras I, Frise E, Kaynig V, Longair M, Pietzsch T, Preibisch S, Rueden C, Saalfeld S, Schmid B, et al: **Fiji: an open-source platform for biological-image analysis.** *Nat Methods* 2012, **9:**676-682.

3. Mayeur N, Minville V, Jaafar A, Allard J, Al Saati T, Guilbeau-Frugier C, Fourcade O, Girolami JP, Schaak S, Tack I: **Morphologic and functional renal impact of acute kidney injury after prolonged hemorrhagic shock in mice.** *Crit Care Med* 2011, **39:**2131-2138.

4. Melnikov VY, Faubel S, Siegmund B, Lucia MS, Ljubanovic D, Edelstein CL: **Neutrophil-independent mechanisms of caspase-1- and IL-18-mediated ischemic acute tubular necrosis in mice.** *J Clin Invest* 2002, **110:**1083-1091.

5. Matute-Bello G, Downey G, Moore BB, Groshong SD, Matthay MA, Slutsky AS, Kuebler WM, Acute Lung Injury in Animals Study G: **An official American Thoracic Society workshop report: features and measurements of experimental acute lung injury in animals.** *Am J Respir Cell Mol Biol* 2011, **44:**725-738.
